# Prospective multicenter cohort study of possible psychogenic nonepileptic seizure cases—Results at 1‐year follow‐up examinations

**DOI:** 10.1002/epi4.12683

**Published:** 2023-01-27

**Authors:** Kousuke Kanemoto, Yukari Tadokoro, Hiromichi Motooka, Jun Kawasaki, Toru Horinouchi, Tomikimi Tsuji, Toshihiko Fukuchi, Oshima Tomohiro

**Affiliations:** ^1^ Epilepsy Center Aichi Medical University Nagakute Japan; ^2^ Kurume University Kurume Japan; ^3^ Kawasaki Mental Clinic Kawasaki Japan; ^4^ Department of Psychiatry & Neurology Hokkaido University Sapporo Japan; ^5^ Department of Neuropsychiatry Wakayama University Wakayama Japan; ^6^ Fukuchi Clinic Kikugawa Japan; ^7^ Present address: Wakayama Tomoda Clinic Wakayama Japan; ^8^ Present address: Koromogahara Hospital Toyota Japan

## Abstract

**Objective:**

The primary purpose of this prospective multicenter study was to examine clinical and demographic feature differences according to the diagnostic level of psychogenic nonepileptic seizures (PNES) and then clarify whether prognosis may also differ accordingly.

**Methods:**

Two hundred forty‐two consecutive patients strongly suspected of having PNES attacks were invited to participate, of whom 52 did not consent or contact was lost. At the 1‐year follow‐up examination, PNES diagnosis was reconsidered in nine patients. In 96 patients, the diagnostic level remained the same (P‐group), with that in 43 considered to be clinically established (CE‐group) and in 42 documented (D‐group). The Qolie‐10 and NDDI‐E questionnaires were examined at both the study entry and the follow‐up examination.

**Results:**

Multiple regression analysis of quality of life (QoL) score (n = 173; *R* ^2^ = 0.374; *F* = 7.349; *P* < 0.001) revealed NDDI‐E score (*t* = −6.402; *P* < 0.001), age of PNES onset (*t* = −3.026; *P* = 0.003), and ethnic minority status (*t* = 3.068; *P* = 0.003) as significant contributors. At entry, the P‐group showed the lowest PNES attack frequency (*P* < 0.000), the lowest rate of antiseizure, antidepressant, and antipsychotic medication (*P* < 0.000; *P* = 0.031; *P* = 0.013, respectively), and the lowest proportion of psychosis (*P* = 0.046). At follow‐up, PNES attack frequency (*P* < 0.000), number of admittances to emergency room (*P* < 0.000), and scores for QoL (*P* < 0.000) as well as depression (*P* = 0.004) were found to be significantly improved together with other collateral indicators, such as rate of antiseizure medication prescription (*P* = 0.001) and psychiatric symptoms (*P* = 0.03). Multiple regression analysis of a sample limited to patients with intellectual disability (ID) (n = 44; *R* ^2^ = 0.366; *F* = 4.493; *P* = 0.002) revealed continued psychotherapy at follow‐up (*t* = 2.610, *P* = 0.013) and successful reduction in antiseizure medication (*t* = 2.868; *P* = 0.007) as positively related with improved QoL.

**Significance:**

Clinical and the socio‐psychological constellation of possible, clinically established, and documented PNES were found to differ greatly. Unexpectedly, significant effects of the continuous psychotherapeutic intervention were confirmed in PNES patients with ID.

**Key Points**

- The first prospective cohort study of possible psychogenic nonepileptic seizure was done.
- Possible psychogenic nonepileptic seizure was less severe than clinically established and documented ones.
- Patients with intellectual disability benefitted from extended psychotherapy.

## 1 INTRODUCTION

It is widely agreed that video‐electroencephalogram (EEG) simultaneous recording is the gold standard for the diagnosis of psychogenic nonepileptic seizures (PNES).^1^, ^2^ Accordingly, most recent prognostic studies concerning PNES are based on exclusively derived from PNES patients with video‐EEG recordings of ictal events. However, in real‐world settings, a substantial proportion of patients with PNES require treatment even without such solid evidence.^3^ Furthermore, physicians in low‐income countries must face the problem of limited accessibility to a video‐EEG monitoring unit,^4^, ^5^ while the successful recording of ictal events usually requires a weekly occurrence of the “seizures” at a minimum, making the resulting sample highly biased, even in high‐income countries, though various helpful methods for inducing PNES “seizures” during the limited time allocated to each patient at a video‐monitoring unit have been suggested.^6^

In this context, the proposal of four diagnostic levels of PNES, possible, probable, clinically established, and documented PNES, suggested by an ILAE task force in charge of PNES^7^ provided the basis for innovative trials. Possible PNES diagnosis is mainly based on history‐taking from patients, family members, and other witnesses of ictal events, along with interictal EEG findings that do not show a corresponding epileptiform discharge. Findings obtained by medical personnel who are not epilepsy specialists and directly observe ictal events are a prerequisite for diagnosing probable PNES, while an epilepsy specialist needs to directly evaluate ictal events to diagnose clinically established PNES. Widespread smartphone availability has greatly increased the chance of recording ictal events and is linked with the dramatic rise of ictal video recordings brought to epilepsy specialists. Documented PNES is identical to that confirmed by simultaneous video‐EEG monitoring, that is, PNES according to the gold standard. It is considered that explicit conceptualization of “possible PNES” may potentially serve as the first step to integrate a thus far discarded portion of PNES cases from samples for scientific investigation back into a prognostic study population.^2^

Although the inclusion of possible PNES in a study sample can be achieved only at the expense of a certain degree of diagnostic accuracy, categorical exclusion of that leads to sampled data that may not represent real‐world PNES cases. Indeed, based on the excellent population‐based study conducted by Villagrán et al.,^8^ the prevalence of PNES including all diagnostic levels was shown to be more than double that of documented PNES. It is considered that both types of data sampling, which certainly supplement each other, are needed. In the present prospective multicenter cohort, the primary purpose was to examine whether clinical as well as demographic features differ according to the diagnostic level of PNES, and then clarify whether prognosis may also differ accordingly. Additionally, we examined whether various therapeutic intervention options can contribute to the improvement of quality of life (QoL) at a 1‐year follow‐up examination. This is the first known prospective cohort study of possible PNES patients after diagnosis.

## 2 SUBJECTS AND METHODS

Two hundred forty‐two patients strongly suspected of having PNES attacks based on examinations by board‐certificated epilepsy specialists at Aichi Medical University, Kurume University, Hokkaido University, Wakayama University, Suzukake clinic, and Kawasaki clinic were consecutively invited to participate in a follow‐up study for a certain period depending on the individual institution from 2016 to 2022, following receipt of written informed consent. Ethical committees of Aichi Medical University, Kurume University, Hokkaido University, and Wakayama University approved this project. Diagnosis of PNES at entry was based on a detailed history taken from patients and others who directly witnessed ictal events, together with interictal EEG findings indicative of no epileptiform discharge. PNES patients with a history of possible additional epileptic seizures were eligible to take part, but only if the two seizure types could be distinguished clearly by the patient or caretaker(s) after diagnosis. In those with co‐morbid epilepsy, a corresponding epileptiform discharge shown by EEG was allowed. An epilepsy specialist explained the high probability of PNES to the patient and, when present, their family members or caretakers. In principle, after explicitly explaining the remaining possibility of mistaking epileptic attacks as PNES, each was recommended to undergo video‐EEG monitoring for recording ictal events or asked to bring a video recording of them taken with a smart phone if video‐EEG monitoring was not used. Some patients without the prospect of prompt arrival at a clinically established or documented level of diagnosis were scheduled for initiation of psychotherapy at the institutions involved in this study, or with a local therapist when the subject already had an existing therapeutic relationship or lived sufficiently far away to make frequent visits logistically difficult simultaneous with continuous efforts to obtain the recording of ictal events. Five patients did not consent to the follow‐up study, while 47 who had consented were lost to contact after entry.

**FIGURE 1:**
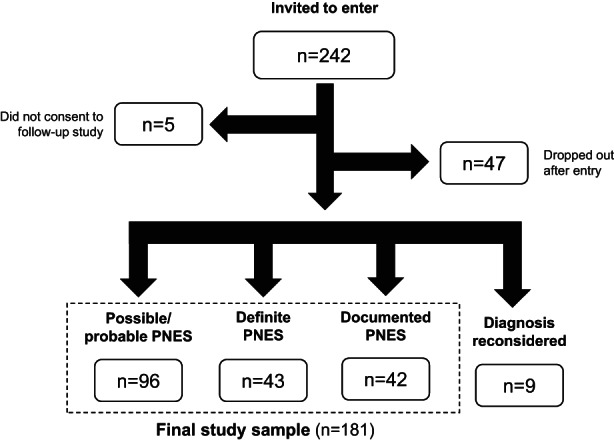
Patient selection flow chart.

Based on the 1‐year follow‐up examination, the diagnosis was converted to epilepsy in eight patients with video or video‐EEG recording of an ictal event. Further, attacks turned out to be a catatonic crisis related to autistic spectrum disorder in additional one patient (the false positive rate is 9.6% [9/(9 + 42 + 43)]). The diagnostic level remained as possible PNES in 96 patients (P‐group), while it was promoted to clinically established in 43 (CE‐group) and documented in 42 (D‐group). Because of difficulties with meaningful separation between probable and possible PNES, probable PNES patients were included with the possible PNES group in the present study. Clinical, demographic, and social findings were accumulated at the time of entry to the study as well as at the follow‐up examination 1 year later. Likewise, the Qolie‐10 and NDDI‐E questionnaires were used on both occasions. In Figure 1, a diagram showing selection of the subjects is presented.

Ninety‐four patients who regularly visited one of the institutions involved in this study were interviewed directly, while the others responded to a package sent by postal mail, including inquiries regarding relevant clinical and socio‐psychological information, as well as two questionnaires. Of the 242 patients invited to participate, 181 (74.8%) were successfully followed at the follow‐up. Patient characteristics of the final study sample were demonstrated in Table 1.

**TABLE 1:** Subjects (*n* = 181)

Gender	109/72
Age at PNES onset, mean [SD]	25.4 [13.5]
Age at PNES diagnosis, mean [SD]	30.2 [14.9]
Years of diagnostic delay, mean [SD]	4.7 [7.2]
Total years of education, mean [SD]	11.8 [2.3]
Traumatic past events or situations	
Child abuse	20 (11.0%)
Ethnic minority	8 (4.4%)
Traumatic event leading to PTSD*	10 (5.5%)
Bullying at school or workplace	67 (37.0%)
Family state at time of entry	
Living with parents	113 (62.4%)
Living alone	11 (6.1%)
Married/cohabitation	50 (27.6%)
Living alone	11 (6.1%)
Married/cohabitation	50 (27.6%)
Employed within past year before entry	78 (43.1%)
Employed at time of entry	
Paid as worker	58 (32.0%)
Sheltered workshop	7 (3.9%)
Housewife or househusband	18 (7.7%)
Student	36 (19.9%)
Old age pension	3 (1.7%)
Welfare program	
Social assistance benefits	11 (6.0%)
Disability pension	18 (9.9%)
Handicapped person's passbook	46 (25.4%)
Welfare support for medical costs	54 (29.8%)
Number of PNES attacks within 3 months before entry	
None	4 (2.2%)
1	16 (8.8%)
2–5	63 (34.8%)
6–10	29 (16.0%)
>10	69 (38.1%)
Number of visits to emergency room within 3 months before entry	
None	89 (49.2%)
1	34 (18.8%)
2–5	50 (27.6%)
>5	8 (4.4%)
Definite triggers	87 (48.1%)
PNES attack symptoms	
Negative motor symptoms	84 (46.4%)
Impaired consciousness	133 (73.5%)
Positive motor symptoms	103 (56.9%)
Duration of PNES attacks immediately before entry	
<5 min	31 (17.1%)
5–30 min	70 (38.7%)
30–60 min	17 (9.4%)
>60 min	63 (34.8%)
Co‐morbid epilepsy	43 (23.8%)
Intellectual disability	50 (27.6%)
Autistic spectrum disorder	24 (13.3%)
Psychiatric symptoms	93 (51.4%)
Dissociative disorder besides PNES	23 (12.7%)
Illness anxiety disorder besides PNES	21 (11.6%)
Somatic symptom disorder	20 (11.0%)
Depressive disorder	18 (9.9%)
Psychosis	13 (7.2%)
Anxiety disorder	7 (3.9%)
Bipolar disorder	2 (1.1%)
Substance‐related disorder	1 (0.6%)
Antiseizure medication administered at time of entry	113 (62.4%)
Psychotropics administered at time of entry	
Antipsychotics	36 (19.9%)
Antidepressants	23 (12.7%)
Benzodiazepines used as psychotropics	36 (19.6%)

### 2.1 Clinical variables

Frequency of attacks obtained from patient reports was subdivided into five divisions based on the number of attacks within 3 months prior to the time of the examination; 0, 1, 2–5, 6–10, and more than 10. The number of visits to an emergency room (ER) within 3 months prior to the time of the examination was likewise subdivided into four divisions; 0, 1, 2–5, and more than 5. The longest duration of PNES within 3 months prior to the examination was also subdivided into four categories; shorter than 5 min, 5–30 min, 30–60 min, and longer than 60 min. Several predominant features of PNES, including positive motor symptoms (rigidity, shaking), negative motor symptoms (weakness, collapse), and impaired consciousness, were noted. Intellectual disability (ID) was defined when a full‐scale IQ score was 75 points or less. The presence or absence of a psychiatric disorder was determined based on DSM‐5 criteria at the time of the examination. Further specifications of any psychiatric disorder were also based on DSM‐5 in principle. However, the presence of a psychotic disorder was simply defined as delusory and/or hallucinatory symptoms confirmed at the time of the examination independent of the DSM‐5 criteria, because delusory and/or hallucinatory states were found not only in the present patients with schizophrenia spectrum disorder but also, more frequently, in those with dissociative disorder. Dissociation and illness anxiety disorder were considered separately from PNES when symptoms different from PNES were present. Neurodevelopmental disorders such as ID and autistic spectrum disorder were considered separately from psychiatric disorder. In the present study, posttraumatic stress disorder was judged to be present only when directly life‐threatening events, such as rape, earthquake, or war, were noted in the history. Patients were regarded as belonging to an ethnic minority when they were the first or second generation of an immigrant family and included Chinese, Korean, Hispanic, Anglo‐Saxon, and Ukrainian origins.

Furthermore, the following therapeutic interventions were examined in view of the impact on the improvement of QoL score; regular visits to epilepsy specialist (n = 93), short‐term psychotherapy including premature discontinuation (n = 60), long‐term psychotherapy continued at the time of follow‐up (n = 44), administration of psychotropics (n = 48), reduction or withdrawal of antiseizure medication (n = 47), environmental adjustment (n = 60), and no therapeutic intervention (n = 46).

### 2.2 Questionnaires

To assess patient QoL, Quality of Life in Epilepsy Inventory‐10 (QOLIE‐10), which has been validated in Japan and translated into Japanese, was applied.^9^ The QOLIE‐10 is a 10‐item inventory designed to determine QoL in adults with consideration that a patient with a diagnosis of epilepsy/seizures has been asked about several aspects related to health over the recent 4 weeks. The overall score is calculated by summing the score of each scale times its weight and then summing all of the scales, with the resultant score converted to a T‐score. This inventory has been previously applied for the evaluation of QoL not only in epilepsy patients but also patients with PNES. To evaluate depression, the Neurological Disorders Depression Inventory for Epilepsy (NDDI‐E) was administered. The NDDI‐E is a self‐administered six‐item screening instrument developed for rapid identification of major depressive episodes in patients with epilepsy and widely used throughout the world,^10^ with the validity and reliability of the Japanese version confirmed by a study conducted by Tadokoro et al^11^ Three patients failed to complete the questionnaire at the time of entry and seven did not return it at 1 year after entry.

### 2.3 Data analysis

Obtained data were analyzed using SPSS version 27.0 for Windows, with *P* values < 0.05 considered to indicate significance. Multiple regression analysis was conducted in order to assess the impact of clinical variables (independent variables) on QoL at entry as well as the time of follow‐up (dependent variable) and also to evaluate the impact of individual therapeutic interventions (independent variables) on the change of QoL between entry and time of follow‐up (dependent variable). The Pearson's correlation coefficient and a *t*‐test were used to select independent variables for regression analysis against QoL. The Mann–Whitney's test and a *t*‐test were applied to compare between patients who did and did not drop out of the study. A Kruskal–Wallis test and ANOVA were applied for comparisons among P‐, CE‐, and D‐groups. Paired t‐test and Wilcoxon signed‐rank test results were used to compare clinical variables between the time entry and at the follow‐up examination.

## 3 RESULTS

### 3.1 Basic clinical and demographic data at entry

The study sample (n = 181) did not differ from the 52 patients (22.3%) who either refused to participate in the follow‐up study or initially consented to the study but were lost to follow‐up in most of the clinical variables except for the proportions of those living alone (17.3% vs 6.1%), who used a psychiatric service (73.1% vs 51.4%), and received administration of an antidepressant (28.8% vs 12.7%), all of which were significantly higher in the drop‐out group, while co‐morbid epilepsy was more often noted in the patients who did not drop out (9.6% vs 23.8%).

In view of QoL score at the time of entry, 15 clinical or psychosocial characteristics were chosen as independent variables for multiple regression analysis as a result of *t*‐test or correlation coefficient (*P* < 0.05). Multiple regression analysis of QoL score at the time entry as a dependent variable (n = 173; *R* ^2^ = 0.374; *F* = 7.349; *P* < 0.001) revealed that NDDI‐E score (*t* = −6.402; *P* < 0.001), age at PNES onset (*t* = −3.026; *P* = 0.003), and belonging to an ethnic minority (*t* = 3.068; *P* = 0.003) were significantly correlated with QoL score. That is, lower QoL at entry was linked with a depressive state and older age at PNES onset. Interestingly, belonging to an ethnic minority was correlated with a higher QoL.

In Table 2, P‐, CE‐, and D‐groups were compared in the 15 clinical characteristics. In summary, PNES attack frequency was lowest in the P‐group (*P* < 0.000). Likewise, antiseizure medication was least frequently prescribed in the P‐group (*P* < 0.000), which remained true even when patients with co‐morbid epilepsy were excluded (*P* < 0.000). Antidepressants and antipsychotics were also least frequently prescribed in the P‐group (*P* = 0.031; *P* = 0.013, respectively), and the proportion of patients with psychosis was also lowest in the P‐group (*P* = 0.046). Handicapped person's passbook and welfare support for medical costs were most frequently noted in the CE‐group (*P* < 0.000; *P* = 0.001, respectively).

**TABLE 2:** Basic clinical and demographic data at entry stratified by diagnostic level

	Possible (*n* = 96)	Clinically established (*n* = 43)	Documented (*n* = 42)	*P* ^*^
Qolie10** (*n* = 176)	34.2 [25.1]	33.4 [27.9]	24.8 [24.6]	0.138
NDDI‐E** (*n* = 176)	15.0 [4.9]	15.0 [5.4]	15.9 [5.1]	0.613
Attack frequency				
None	4 (4.2%)	0 (0%)	0 (0%)	
1	14 (14.6%)	0 (0%)	2 (4.8%)	0.000^†^
2–5	36 (37.5%)	14 (31.6%)	13 (31.0%)	
6–10	15 (15.6%)	6 (14.0%)	8 (19.0%)	
>10	27 (28.1%)	23 (53.5%)	19 (45.2%)	
Delay of diagnosis (yrs.)	4.0 [6.2]	5.7 [8.3]	5.8 [7.7]	0.248
Age at PNES onset (yrs.)	24.4 [11.7]	25.4 [15.2]	28.0 [15.6]	0.354
Age at entry (yrs.)	28.3 [13.0]	31.1 [16.7]	33.8 [16.7]	0.131
Co‐morbid ID	14 (14.6%)	18 (41.9%)	18 (42.9%)	0.001^†^
Co‐morbid epilepsy	17 (17.7%)	15 (34.9%)	11 (26.2%)	0.083
Ethnic minority	5 (5.2%)	1 (2.3%)	2 (4.8%)	0.742
Psychosis	3 (3.1%)	6 (14.0%)	5 (11.9%)	0.046^†^
Antiseizure medication	44 (45.8%)	36 (83.7%)	34 (81.0%)	0.000^†^
***	27/79 (34.2%)	21/28 (75.0%)	23/31 (74.2%)	0.000^†^
Benzodiazepine as a psychotropic	14 (14.6%)	13 (30.2%)	8 (19.0%)	0.098
Antipsychotic medication	13 (13.5%)	14 (32.6%)	10 (23.8%)	0.031^†^
Antidepressant medication	9 (9.4%)	4 (9.3%)	11 (26.2%)	0.019^†^
Handicapped person's passbook	12 (12.5%)	21 (48.8%)	12 (28.6%)	0.000^†^
Welfare support for medical costs	20 (20.8%)	22 (51.2%)	11 (26.2%)	0.001^†^

Abbreviation: ID, intellectual disability

* Statistical analysis conducted by Kruskal–Wallis test or ANOVA, based on data type.

** Because of failure to complete questionnaire, five patients were not included in the analysis.

*** Number indicates proportion given to patients without epilepsy.

^†^ Statistically significant difference.

### 3.2 Outcome at 1‐year follow‐up

As shown in Table 3, the major indicators of the severity of PNES, attack frequency (*P* < 0.000), delivery to ER, QoL (*P* < 0.000), and scores for QoL (*P* < 0.000) and depression (*P* = 0.004) at the 1‐year follow‐up examination were found to be significantly improved. Except for employment status (*P* = 0.132), other collateral indicators, that is, rate of prescription of antiseizure medication (*P* = 0.001) and psychiatric symptoms (*P* = 0.03), were also significantly diminished. When stratified by diagnostic level, QoL, attack frequency, and visits to ER were most favorable in the P‐group (*P* = 0.019; *P* < 0.000; *P* < 0.000, respectively) (Table 4). In the P‐group, 30 patients had stopped antiseizure medication without recurrence of seizures.

**TABLE 3:** Outcomes at 1 year after entry stratified by diagnostic level (*n* = 181)

	Entry	One year after entry	*p* *
Qolie10** (*n* = 171)	31.9 [25.9]	49.1 [29.7]	0.000***
NDDI‐E** (*n* = 171)	15.1 [5.1]	14.1 [5.2]	0.004***
Attack frequency			
None 1 2–5 6–10 >10	4 (2.2%) 15 (8.3%) 63 (34.8%) 29 (16.0%) 70 (38.0%)	89 (49.2%) 20 (11.0%) 34 (18.8%) 13 (7.2%) 25 (13.8%)	0.000***
Visits to ER			
None 1 2–5 >5	88 (48.6%) 34 (18.8%) 50 (27.6%) 9 (5.0%)	158 (87.3%) 12 (6.6%) 10 (5.5%) 1 (0.6%)	0.000***
Antiseizure medication	116 (63.4%)	68 (37.2%)	0.001***
Psychiatric symptoms	95 (52.2%)	78 (43.1%)	0.03***
Employed	79 (43.4%)	89 (48.9%)	0.132

* Statistical analysis conducted by paired t test or Wilcoxon signed‐rank test, based on data type.

** Because of failure to complete questionnaire, 10 patients not included in analysis.

^***^ Significant difference.

**TABLE 4:** Outcomes at 1 year after entry stratified by diagnostic level

	Possible (*n* = 96)	Clinically established (*n* = 43)	Documented (*n* = 42)	*p* *
Qolie10 ** (*n* = 176)	55.3 [29.6]	43.8 [30.9]	40.3 [26.7]	0.019†
NDDI‐E ** (*n* = 176)	13.4 [5.3]	14.6 [5.3]	15.3 [4.9]	0.077
Attack frequency				
None 1 2–5 6–10 >10	60 (62.5%) 11 (11.6%) 15 (15.8%) 4 (4.2%) 5 (5.3%)	15 (34.9%) 4 (9.3%) 10 (23.3%) 4 (9.3%) 10 (23.3%)	12 (31.0%) 5 (11.9%) 9 (21.4%) 5 (11.9%) 10 (23.8%)	0.000†
Visits to ER				
None 1 2–5 >5	88 (91.6%) 5 (15.8%) 2 (2.1%) 1 (1.0%)	38 (88.1%) 1 (2.3%) 4 (9.3%) 0 (0.0%)	32 (76.2%) 6 (14.3%) 4 (9.5%) 0 (0.0%)	0.043†
Antiseizure medication	30 (31.3%)	20 (46.5%)	18 (42.9%)	0.167
Psychiatric symptoms	35 (36.5%)	22 (51.2%)	21 (50.0%)	0.036
Employed	51 (53.1%)	22 (51.2%)	16 (38.1%)	0.257

* Statistical analysis conducted by Kruskal–Wallis test or ANOVA, based on data type.

** Because of failure to complete the questionnaire, 10 patients were excluded from analysis.

^†^ Statistically significant difference.

### 3.3 Evolution of QoL as function of therapeutic intervention

Although multiple regression analysis of QoL change, that is, the value for QoL at entry subtracted from that at follow‐up as a dependent variable and the value for individual therapeutic interventions as independent variables, failed to produce a meaningful linear regression model (n = 168; *R* ^2^ = 0.058; *F* = 2.022; *P* = 0.078), multiple regression analysis in the sample limited to patients with ID (n = 44; *R* ^2^ = 0.366; *F* = 4.493; *P* = 0.002) revealed that long‐term psychotherapy still continued at the time of the examination (*t* = 2.610, *P* = 0.013) as well as successful reduction of antiseizure medication (*t* = 2.868; *P* = 0.007) were positively related with improved QoL. Furthermore, short‐term psychotherapy including premature discontinuation was inversely related with QoL improvement (*t* = −2.477; *P* = 0.018).

## 4 DISCUSSION

The present results clearly demonstrated that the clinical and socio‐psychological constellation of P‐, CE‐, and D‐groups differs greatly. Major clinical variables indicative of its severity, that is, high attack frequency and frequent visits to an ER, were more frequent in the CE‐ and D‐groups as compared to the P‐group. While antiseizure medication was noted to have been prescribed by a prior doctor at the time of entry in the majority of the CE‐ and D‐groups, less than half of the patients in the P‐group had received such an administration. This was also true even when patients with co‐morbid epilepsy were excluded from analysis. Additionally, the proportion of patients with ID or psychosis was much higher in the CE‐ and D‐groups than in the P‐ group. Overall, possible PNES was revealed as a milder variant of PNES in regard to severity.

Previous studies have suggested that QoL is directly correlated with the degree of depressive state but not with PNES attack frequency,^12^, ^13^, ^14^ which was confirmed by the present findings. While NDDI‐E score proved to be closely correlated with QoL in all of the diagnostic levels, no correlations between attack frequency and QoL at the time of entry were found. Also, younger age at PNES onset proved to be associated with high QoL score in the present study, in agreement with previous studies, which have consistently linked better PNES condition with younger age at onset.^15^, ^16^, ^17^, ^18^ Thus, a different underlying psychopathological mechanism is proposed as a possible explanation.^19^ That is, PNES in children and adolescents may be more related to a reaction to transient stress, while chronic psychological maladjustment such as personality disorder may be more relevant in adults.^20^ In the present study, belonging to an ethnic minority was associated with high QoL. As with children and adolescents, this might also be explained as a transient psychological reaction to a stressful environment in psychologically healthy individuals. Although the heightened likelihood of nonadherence to psychotherapy by this subgroup demonstrated by Tolchin, et al.^21^ seems to superficially conflict with the present data, it might not be in reality, in view of the possible predominance of patients with good prognosis among those who dropped out.^22^, ^23^

**FIGURE 2:**
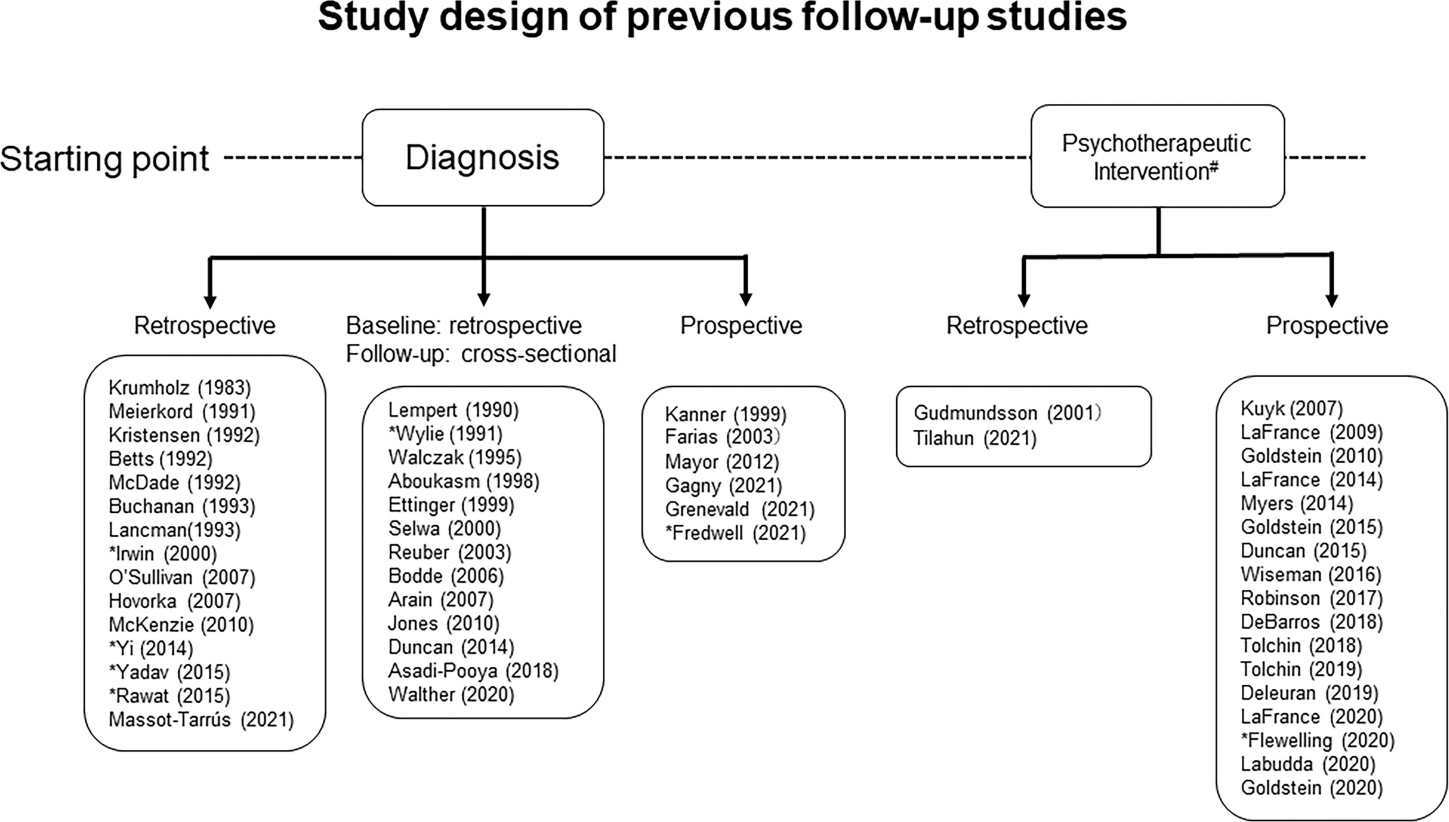
Overview of the previous stuides on this topic is provided. *Reports focused on children or adolescents. #The reports listed in this category are not included in the reference list.

A large number of follow‐up studies regarding PNES have been published since the 1990 s and can be divided based on how the starting point was determined, as summarized in Figure 2. While the time of diagnosis was mostly used as the starting point in the early observational studies,^16^, ^19^, ^20^, ^23^, ^24^, ^25^, ^26^, ^27^, ^28^, ^29^, ^30^, ^31^, ^32^, ^33^, ^34^, ^35^, ^36^, ^37^, ^38^, ^39^, ^40^, ^41^, ^42^, ^43^, ^44^, ^45^, ^46^, ^47^, ^48^, ^49^, ^50^ more recent studies often start at the point of psychotherapeutic intervention (Figure 2). As for the former type, while follow‐up data were newly inquired in half of those,^16^, ^19^, ^23^, ^25^, ^32^, ^33^, ^35^, ^36^, ^38^, ^42^, ^45^, ^49^ baseline data were acquired by a retrospective examination of case records, except for a few exceptions.^34^, ^37^, ^39^, ^50^ However, even with those exceptions, the prospective nature of the study design was highly limited in some, either because of the extreme shortness of the follow‐up period (24 h),^39^ or an obscure distinction between baseline and follow‐up data.^33^ The study of Mayor et al.^37^ was done with a strictly prospective design, though the responder rate remained at 40%. The most recently published studies presented by French investigators were also strictly prospective. However, direct comparisons between baseline and follow‐up data were not presented, because they focused on the evolution of seizure frequency^51^ or QoL.^14^ Additionally, a prospective study of child patients published recently, which included cases of possible PNES as in this study, adopted attack frequency as the sole measure of PNES outcome.^50^ In this context, the use of a prospective cohort such as in the present investigation has been rarely done among follow‐up studies that used the time of diagnosis as the entry point.

Overall, the major clinical outcomes shown in the former type of follow‐up studies have been discouraging. In both short‐ and long‐term studies, the average rate of PNES cessation is generally reported to be low, ranging from 13% to 55%,^16^, ^18^, ^23^, ^25^, ^26^, ^27^, ^29^, ^30^, ^31^, ^32^, ^33^, ^35^, ^36^, ^37^, ^38^, ^40^, ^41^, ^42^, ^43^, ^44^, ^45^, ^47^, ^49^, ^50^ except for a few reports among those exclusively dedicated to children and adolescents,^18^, ^19^, ^46^, ^48^ with the rates in those ranging from 66–83%. The seizure cessation rate in the present study (48.9%) was close to the upper limit in previous studies. Psychiatric symptoms have also been utilized as an important measure to indicate the severity of PNES. While prevalence greatly varies among individual studies,^18^, ^23^, ^31^, ^32^, ^34^, ^35^, ^41^, ^43^, ^45^, ^48^, ^49^ ranging from 26% to 77%, either data at follow‐up or at entry are lacking in most of those. Two studies reported the prevalence of both at entry and at follow‐up. Duncan, et al.^23^ reported a slightly increased prevalence of psychiatric contact at the follow‐up examination (26.5%) as compared with that at entry (22.9%), while Jones, et al.^45^ reported a much higher prevalence of psychiatric symptoms at both the follow‐up (72.9%) and entry (63.8%) points. In both of those studies, no statistically significant improvement of psychiatric symptoms or involvement of a psychiatric service was confirmed. Although the present result is considered to be intermediate (43.1%) in view of the prevalence of psychiatric comorbidity, any direct comparison is difficult due to the different methods of psychiatric assessment used. However, different from other studies, our results demonstrated a significant decrease in psychiatric symptoms at the follow‐up examination. As for employment, other studies have noted that the proportion of patients actively working at the time of follow‐up was also relatively low,^16^, ^18^, ^23^, ^25^, ^34^, ^35^, ^38^, ^40^, ^42^, ^43^, ^44^, ^45^, ^49^ ranging from 22% to 50%, except for the exceptionally low 5% reported by McDade et al.^27^ In view of improvement of employment status, some studies denied^44^ while others confirmed that.^16^, ^23^ The rate of active employment in the present study at follow‐up (48.9%) was slightly greater than the upper extreme noted in previous studies, though a statistically significant improvement was not confirmed. The observational period of the present study might be too short to improve the employment status. In view of QoL, structured psychosocial assessments were mostly lacking at the initial examination in previous studies, except for some exceptions.^14^, ^37^, ^40^ While Bodde et al.^40^ demonstrated a positive evolution of QoL, in agreement with the present study, Mayor et al.^37^ denied that. Overall, except for employment, all major clinical variables indicative of the severity of PNES in the present study were shown to be improved. Altogether, the clinical outcomes seen in the present cohort, which included possible PNES, belonged to the least unfavorable ones as compared to previous studies, although the potential contribution of some patients included in the “possible PNES” cohort with other disorders that had a good prognosis must be considered as an important alternative explanation.

Although multiple regression analysis of QoL evolution in response to therapeutic intervention failed to produce a meaningful model as a collective sample in the present study, continued psychotherapeutic intervention as well as successful reduction of antiseizure medication at the time of follow‐up were shown to be associated with positive QoL evolution when the analysis was focused on subjects with ID. By contrast, it is interesting to note that psychotherapeutic interventions that had already been terminated at the time of follow‐up, such as short‐term psychotherapeutic intervention as well as premature discontinuation of that, were associated with a negative evolution of QoL. This agrees with the results that Gagny et al.^14^ noted regarding the effects of the number of mental health consultations on the positive evolution of QoL. Furthermore, Tilahun, et al.^52^ demonstrated that an extended period of psychotherapeutic intervention had potential efficacy for seizure control, depression, and anxiety, even with the same number of sessions, though the change in QoL did not reach a level of statistical significance in their sample. In most previous studies of PNES that investigated the efficacy of psychotherapeutic intervention, patients with ID were systematically excluded from analysis. This subgroup of PNES patients tends to develop chronic PNES and become heavy users of medical resources.^3^ Two groups of authors who have focused on PNES in patients with ID have advocated a “reinforced behavioral pattern” as a strong pathogenetic factor and emphasized the need for a specific therapeutic approach.^53^, ^54^ Certainly, the negative impact of premature termination of psychotherapeutic intervention allows for multiple interpretations, including a poor prognosis in patients who do not engage well with psychotherapeutic interventions or who terminate them as well as a nonspecific effect on the quality‐of‐life of the intervention independent from the effects of psychotherapeutic intervention. Nevertheless, together with the study by Gagny et al.,^14^ the present data suggest that continuous involvement in mental health consultations by patients for an extended period can improve QoL, especially those in this subgroup, thus supporting the proposal of those authors. In view of the prevailing trend of exclusion of this subgroup of PNES patients from psychotherapeutic intervention, the present results should be noted and reappraised, possibly leading to reconsideration of the exclusion of PNES patients with ID from a psychotherapeutic approach. Additionally, in agreement with previous studies,^32^, ^33^ a reduction in antiseizure medication was also associated with the positive evolution of QoL in this subgroup, though it remains to be determined whether a reduction of antiseizure medication improved QoL by alleviating toxic side‐effects or psychosocial recovery leading to the cessation of PNES attacks enabled that reduction.

The inclusion of possible PNES in the present analysis might be considered a limitation, though it is also thought of as a strong point. It is likely that both epileptic and seizure‐like symptoms of organic origin were included in the analysis, which may obscure conclusions to be drawn from our data. The findings that attacks were less frequent might have been due wholly, or at least in part, to the inclusion of patients with such conditions as mild epilepsy or syncope, as those are likely to have a low attack frequency. However, based on a simple calculation, the false positive rate of diagnosis in the present possible PNES group might have been less than 10%. Even if 30% of the diagnoses would need to be reconsidered, based on the average false positive rate of PNES diagnosis,^55^ 79% of the diagnoses are assumed to be true when patients who remained seizure free even after withdrawal of antiseizure medication are allowed to be regarded as having PNES. The diagnostic accuracy of clinically established PNES may also be doubted,^56^ though high reliability of diagnosis was recognized in this group of PNES patients in other reports.^57^ Nevertheless, if the severity and prognosis of PNES differ according to different diagnostic levels, as the present results demonstrate, then observational follow‐up studies inclusive of a possible PNES group are mandatory to obtain a more complete view of PNES and provide important supplementary data to documented cases. Additionally, both questionnaires used in the present study, NDDI‐E and Qolie‐10, are simplified versions of more extensive rating scales used to assess depression and QoL, respectively. Many previous studies, especially those that started at the point of psychotherapeutic intervention, used more detailed and extensive types of questionnaires, as well as multiple psychological tests. However, as Tolchin et al. noted,^20^ the adherence of PNES patients to medical service in real‐world practice is astonishingly low, as they found that only 14% of those patients remained committed through the fourth visit. With the use of more extensive and time‐demanding questionnaires, especially in observational studies that start at the time of diagnosis, nonadherence is more likely to be encountered.

In conclusion, results obtained in the present study may shed new light on the prognosis of PNES patients and supplement data derived from documented PNES cases. Confirmation by further studies is urgently needed.

## CONFLICT OF INTEREST

None of the authors has any conflict of interest to disclose. We confirm that we have read the Journal's position on issues involved in ethical publication and affirm that this report is consistent with those guidelines. All listed authors have contributed to the manuscript substantially and have agreed to the final submitted version.
